# Probiotics: A Potential Strategy for Preventing and Managing Cardiovascular Disease

**DOI:** 10.3390/nu17010052

**Published:** 2024-12-27

**Authors:** Anallely López-Yerena, Victoria de Santisteban Villaplana, Lina Badimon, Gemma Vilahur, Teresa Padro

**Affiliations:** 1 Institut Recerca Sant Pau, Sant Quinti 77-79, 08041 Barcelona, Spain; naye.yerena@gmail.com (A.L.-Y.); vsantisteban@santpau.cat (V.d.S.V.); lina.badimon@gmail.com (L.B.); gvilahur@santpau.cat (G.V.); 2 School of Pharmacy and Food Sciences, University of Barcelona (UB), 08036 Barcelona, Spain; 3 Centro de Investigación Biomédica en Red Cardiovascular (CIBER-CV), Instituto de Salud Carlos III, 28029 Madrid, Spain; 4 Cardiovascular Research Foundation for Health Prevention and Innovation (FICSI), 08017 Barcelona, Spain

**Keywords:** inflammation, lipid profile, functional foods, blood pressure

## Abstract

Probiotics are gaining recognition as a viable strategy for mitigating cardiovascular risk factors. Specifically, recent studies highlight their potential benefits in managing cholesterol levels, blood pressure, and inflammation, which are critical components in the prevention of cardiovascular diseases (CVD). This comprehensive review aims to elucidate the impact of probiotic consumption on major cardiovascular risk factors, including individuals with hypertension, type II diabetes mellitus, metabolic syndrome, hypercholesterolemia, and in secondary prevention in coronary artery disease. Scientific evidence based on human studies suggests that probiotic consumption is associated with positive effects on anthropometric measures, inflammation markers, blood pressure, glucose metabolism markers, lipid profiles, and endothelial function. However, these findings should be interpreted pragmatically and acknowledge the significant variability in results. This variability may be attributed to factors such as probiotic composition (single strain or multiple strains), the characteristics of the delivery matrix (food, capsules, and sachets), the duration of the intervention, the dosage regimen, and baseline health profiles of the participants. Incorporating probiotics as part of a comprehensive and healthy lifestyle approach can be considered a feasible strategy for both the prevention and management of CVD. However, further research is needed on factors influencing the effect of probiotics, such as: (i) optimal probiotic strain(s), (ii) appropriate dosage, (iii) duration of treatment, (iv) optimal delivery vehicle, and (v) sex-specific differences.

## 1. Introduction

Since its appearance in 1908 by Metchnikoff [1], the term probiotic has been associated with host health benefits. In 2001, The World Health Organization (WHO) and the Food and Agriculture Organization of the United Nations (FAO) considered probiotics as live micro-organisms, which when administered in adequate amounts confer a health benefit on the host [2]. In 2014, a consensus panel of the International Scientific Association for Probiotics and Prebiotics (ISAPP) reaffirmed the definition with one minor change: replacing “which” with “that”, resulting in “live microorganisms that, when administered in appropriate amounts, confer a health benefit on the host” [3].

To date, numerous studies highlight the fact that, in addition to the antipathogenic, anticancer, antiallergic, and angiogenic activity [4], the probiotic health implications include protection against cardiovascular diseases (CVDs) [5]. Despite milestones in preventive measures and treatment, CVD remains associated with a high burden of morbidity and mortality. There are several established risk factors for CVD that include hypertension, dyslipidemia, diabetes, obesity, smoking, alcohol, diet, and sedentary lifestyle [6]. The gut microbiome has been causally linked with cardiometabolic and cardiovascular disorders and is a potential complementary target to understand and reduce the residual risk of CVD [7]. In fact, the comprehensive characterization of probiotic benefits on cardiovascular risk factors has demonstrated the glycemic control in patients with type two diabetes mellitus (T2DM) [8], improvement in inflammatory markers in coronary artery disease (CAD) patients [9] and in obesity-related markers in obese people [9], among others. In fact, probiotic consumption in healthy populations has recently been shown to be associated with increased protection against atherosclerotic disease [10]. As diet-related risk is the most important behavioral factor influencing global health, it appears to be the best target in the challenge against CVD [11].

The gut microbiota has the capability to generate metabolic products from dietary sources that have the potential to exert an influence on the cardiovascular health of the host [12]. Simultaneously, the consumption of probiotics is accompanied by significant alterations in the composition of the intestinal microbiota [13,14,15,16,17]. Furthermore, in tandem with all of the aforementioned factors, emerging risk factors, such as pro-inflammatory molecules (e.g., C-reactive protein (CRP), interleukins (IL)), adhesion molecules, apolipoproteins, glucose metabolism markers (C-peptide, insulin, hemoglobin A1c (HbA1c)), and cholesterol fractions, have been analyzed and linked to an increased cardiovascular risk, prognosis, and complications of CVD [18] and therefore have also been targeted in clinical interventions trying to ensure the effect of probiotic consumption on these markers.

Although the effect of probiotics on CVD has been summarized in numerous papers, most of these have focused on a target population such as T2DM [19], CVD risk patients [20], or in specific markers [21]. Additionally, existing reviews often evaluate not only the effects of probiotics but also include prebiotics and symbiotics, resulting in a broader and less focused analysis [22,23]. In this comprehensive review, we examine the impact of probiotic consumption on vulnerable populations, including individuals with hypertension, T2DM, metabolic syndrome (MetS), hypercholesterolemia, and in secondary prevention in CAD. For this purpose, only studies conducted in humans since 2015 to date and in the English language were included. Scopus, Pubmed, and Google Scholar were used to search for clinical trials testing the effect of probiotics in individuals with hypertension, T2DM, MetS, hypercholesterolemia, and CAD. The search initially identified a total of 186 studies. However, after applying the inclusion criteria—human studies, English language, and probiotics administered alone (not co-administered)—only 44 articles met the criteria and were included in this review.

## 2. Probiotics

**Figure 1:**
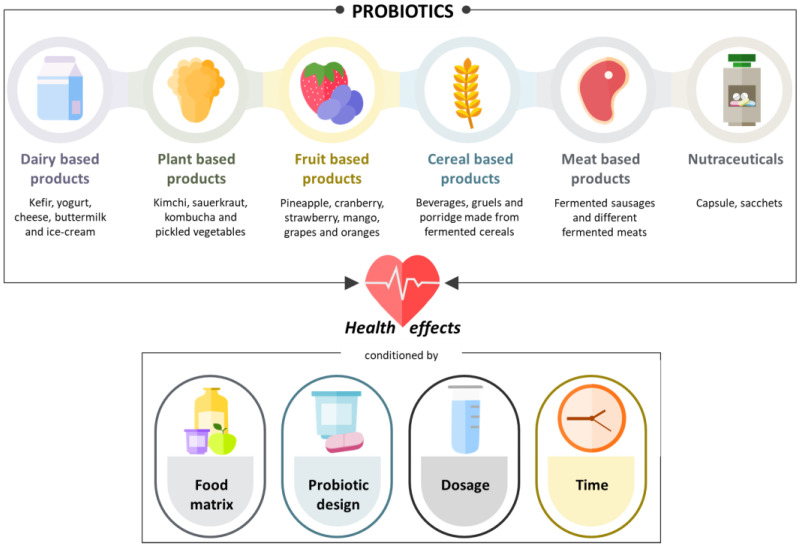
Main probiotic forms.

Probiotics are essential functional foods that play a crucial role in maintaining and improving overall health and well-being. Oral probiotics face several challenges on their journey through the mouth, stomach, intestine, and colon. In specific, many microbial species are adversely affected by harsh gastrointestinal conditions characterized by low pH in the stomach and the presence of bile salts in the small intestine [24,25]. Therefore, one of the most important requirements for probiotics is to survive the environmental conditions in the location where they must be active. Successful colonization of the gastrointestinal tract is key to ensuring that probiotics can interact with the host to achieve the desired health benefits [26]. To date, numerous studies have highlighted all those factors that benefit or limit the effect of probiotics. Food matrix [27,28], probiotic design [29], and doses [30], among others, have a recognized effect (Figure 1).

Probiotic design (monostrain, multistrain, and multispecies probiotics) is raised as an important consideration that can affect the functionality and efficacy of a probiotic [29]. Studies using animal models have shown that multistrain probiotics are more efficient in obesity management [31]. However, to date, very little is known about which preparation has the greatest effect on preventing CVD.

A systematic review and meta-analysis of clinical trials evidenced that the effectiveness of the probiotic is conditioned by the dosage [32]. Regulatory agencies around the world recommend minimum dosages. For example, Health Canada has accepted bacterial species when delivered in food at a level of 1 × 10^9^ colony-forming unit (CFU) per serving, and the Italian Ministry of Health confirmed the use of the word probiotic for food and food supplements under certain conditions, including a minimum number of viable cells (1 × 10^9^ CFU) administered per day [33,34]. However, recent reports have indicated that the dose required for most strains to survive and persist is higher (1 × 10^10^ CFU) (1 × 10^10^ CFU) than the doses recommended for probiotics by regulatory agencies [35]. Similarly, in obese postmenopausal women, multispecies probiotic supplementation favorably affects vascular function and reduces arterial stiffness when given at the dose of 1 × 10^10^ CFU per day [36].

The effect of the probiotics is also conditioned by the time of the intervention [37]. Regarding patients with risk factors for CVD, there is no trend as to whether short- or long-term interventions are more advisable [38].

### Foods

Foods are vehicles for delivery of probiotics to the human body and help to buffer the probiotics through the gastrointestinal tract, regulate their colonization, and contain other functional ingredients, such as bioactive components, that can interact with probiotics to alter their functionality and efficacy [28]. In foods, probiotics may be present in fermented dairy products (e.g., yogurt, cheese, and fermented milk), which represent the main vehicle for probiotic delivery (Figure 1). Additionally, many other nondairy probiotic products (both fermented and nonfermented) are gaining popularity among consumers. Currently, a wide range of nondairy probiotic foods on the market, such as soy products, cereal-based products, fruit and vegetable juices, and fermented meat and fish products, are also offered [39,40]. To date, cheese, yogurt, milk (soy, probiotic, or fermented), kefir, and light Yakult are by far the main food vehicles used for the delivery of probiotics.

However, it is important to note that the delivery of probiotics to the host can also be accomplished through other alternatives. In specific, capsules are also a growing trend to use probiotics as nutraceuticals [41]. However, it is important to emphasize that this changing trend in probiotic delivery may result in reduced functional efficacy by eliminating the potential synergistic effect of the food. Probiotics in sachets or capsules are commonly used in studies evaluating their effect in patients at risk of CVD.

## 3. The Health-Promoting Properties of Probiotics in the Management and Prevention of CVD

There are several established risk factors for CVD that include hypertension, hypercholesterolemia, T2DM, overweight and obesity, smoking, and a sedentary lifestyle [42,43]. Among these risk factors, high blood pressure is associated with the strongest evidence of causality and has a high prevalence of exposure [44]. In recent decades, a growing number of emerging risk factors, such as proinflammatory molecules including CRP, interleukins (IL), adhesion molecules, apolipoproteins, and cholesterol fractions, among others, have been analyzed and associated with increased cardiovascular risk, CVD prognosis, and complications [18].

The relationship between diet and CVD has been the focus of attention for some time. From this perspective, there is growing evidence that functional dietary intervention with probiotics, which maintains or restores the beneficial bacteria of the digestive tract, is a promising therapeutic strategy for intervention in CVD and also reduces the risk of its occurrence [45]. Recently, it was demonstrated that the intervention with *L. plantarum* strains induces beneficial effects on bile acid signature and lipoprotein profile. It reduces ApoB and small LDL levels and LDL susceptibility to oxidation and increases HDL antioxidant capacity in healthy individuals [10].

**Figure 2:**
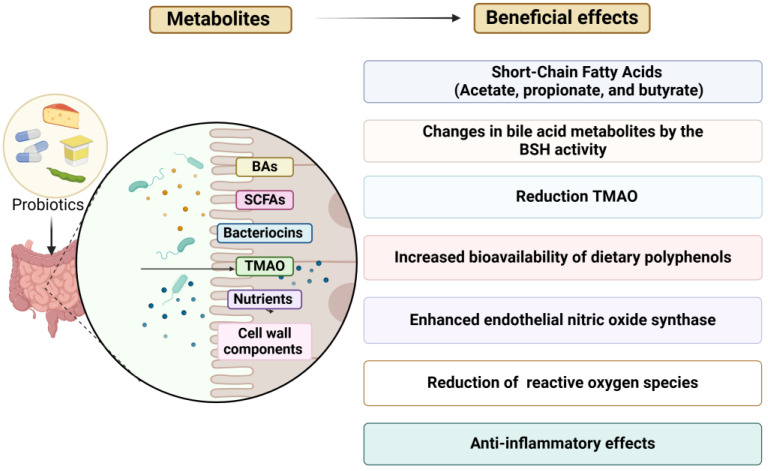
Key metabolites that act as potential messengers in the molecular and functional processes that mediate the health-promoting effects of probiotics.

The following sections summarize the evidence for the effect of probiotic consumption on risk factors for CVD. Figure 2 highlights the key metabolites that act as potential messengers in the molecular and functional processes mediating these beneficial effects. These include short-chain fatty acids (SCFAs) such as acetic, propionic, and butyric acids; bacteriocins with antimicrobial properties; vitamins and essential nutrients (e.g., vitamin K, B vitamins, and aromatic amino acids); organic acids; and cell wall components with immunomodulatory effects [46]. SCFAs play vital roles in regulating the immune system, maintaining gut barrier integrity, improving endothelial function, and modulating cholesterol and glucose metabolism [47]. Although bile acids (BAs) are not directly microbiota-derived, the gut microbiota significantly influences their transformation, playing a key role in cholesterol catabolism [48].

### 3.1. Hypertension

Hypertension is a leading risk factor for mortality worldwide, accounting for 10.8 million deaths in 2019. Hypertension or high blood pressure, defined as systolic blood pressure (SBP) above 140 mmHg and diastolic blood pressure (DBP) above 90 mmHg in young people, is one of the most important risk factors that predispose an individual to many diseases, including coronary heart disease, cerebral hemorrhage, renal and cardiac failure [49]. Blood pressure is controlled by several complex biochemical pathways. The renin-angiotensin system (RAS) is identified as one of the major controllers of blood pressure and sodium metabolism. In addition, the kinin-nitric oxide, the neutral endopeptidase, and the endothelin-converting enzyme systems have been shown to produce additional vasoregulatory peptides [50].

Probiotics and their fermented products reduce blood pressure by suppressing nitrogen oxide production in the microphages, reducing reactive oxygen species, and improving the absorption of calcium from the diet [51]. More specifically, probiotics enhance endothelial nitric oxide synthase (eNOS) activity, increasing NO bioavailability and promoting vasodilation, which reduces vascular resistance and blood pressure [52,53]. Probiotics also lower oxidative stress and inflammation, protecting NO from degradation and preventing endothelial dysfunction [54]. Additionally, they improve L-arginine availability [55], a key substrate for NO synthesis. Through these mechanisms, probiotics help regulate vascular tone, reduce arterial stiffness, and maintain healthy blood pressure levels, highlighting their therapeutic potential in hypertension management.

A cross-sectional study demonstrated that the exposure to probiotics resulted in a 21% reduction in the odds of hypertension that was evidenced by a significant reduction in SBP and DBP [56]. The rest of the evidence is based on systematic review and meta-analysis focused on human studies [37,57,58,59,60]. Despite the enormous concern about the CVD problems associated with hypertension, only five human interventional studies testing the probiotic effects [60,61,62,63,64]. The characteristics of these clinical studies, as well as their main findings, are summarized in Table 1. Of these five studies analyzed, the beneficial effect of probiotic intake on SBP and DBP was evidenced in only two studies of four [61] and eight weeks [60] after regular consumption of cheese with *L. casei* 01 or multistrain capsule. On the other hand, both SBP and DBP remained unchanged in six- [64], eight- [62], and twelve- [63] week studies.

### 3.2. Type 2 Diabetes Mellitus (T2DM)

T2DM is a chronic, non-communicable, multisystem disease that has reached epidemic proportions [65]. T2DM is characterized by dysfunction in pancreatic β-cells, chronic low-grade inflammation, oxidative stress, insulin resistance, and dysregulation of lipid and glucose metabolism [66]. Oxidative stress significantly impacts β-cell health and function in T2DM through mechanisms involving ROS production, antioxidant deficiency, and interactions with metabolic dysregulations, such as insulin resistance and lipotoxicity [67,68]. Addressing these pathways may offer new avenues for diabetes treatment aimed at preserving β-cell function and improving glycemic control.

Multiple molecular mechanisms of gut microbiota contribution to T2DM have recently been summarized, including: (i) modulation of inflammation, (ii) interaction with dietary components, (iii) involvement of intestinal permeability, and (iv) involvement of glucose and lipid metabolism, insulin sensitivity, and overall energy homeostasis in the mammalian host [69,70]. In view of the high incidence of T2DM and its serious consequences, from 2015 to date, a series of human trials have been conducted to evaluate the efficacy of probiotic consumption in patients with this pathology. Specifically, these studies have focused on assessing the effect of probiotic soya milk [71,72,73], fermented milk [74], or through sachets [75,76,77,78] or capsules [38,79,80,81,82] on several biomarkers, as is shown in Table 2.

As for anthropometric measures, 50% of the 14 studies included in this review assessed these parameters (Table 2). In a study testing soy milk fortified with *L. plantarum* A7, eight weeks of sustained intake led to a decrease in weight, BMI, and waist−hip ratio [73]. In another study, eight weeks of multiprobiotic “Symbiter” (concentrated biomass of 14 probiotic bacteria genera *Bifidobacterium*, *Lactobacillus*, *Lactococcus*, *Propionibacterium*) administered as a sachet formulation led to a decrease in weight, BMI, and waist circumference [77]. In addition, another study showed that the dose (low vs. high) affects the anthropometric measurements differently, in particular, an increase in weight and BMI was observed in the low group [76]. The remaining studies found no evidence that probiotic consumption induced changes in anthropometric measures in T2DM populations.

Regarding changes in the glycemic profile, the following findings were reported. Importantly, a decrease in FBG levels was observed when the probiotic was administered in capsule or sachet forms [75,78,79,80,81]. A similar behavior was observed for HOMA-IR, where a decrease was observed when the probiotic was administered mainly in the sachets, regardless of the duration of the study (Table 2). These findings may be directly related to the fact that probiotics administered in capsule or sachet form are usually multistrain compared to foods that are mostly monostrain. Both monostrain and multistrain probiotics have their unique advantages and limitations. However, in the glycemic control of patients with T2DM, evidence would suggest that the multistrain probiotics provide a broader range of benefits through synergistic effects. The level of HbA1c in T2DM patients can accurately reflect the patient’s glycemic control [83]. However, there is no pattern of response to probiotic use, with one study showing an increase, three showing a decrease, and three showing no change (Table 2). Another key feature of T2DM is circulating insulin, but in most of the studies evaluating this biomarker, no changes in its concentration were observed. The same trend was observed for C-peptide.

The pathophysiology of T2DM reveals that oxidative stress is one of the factors that play a role in the pathogenesis of insulin resistance, impaired insulin secretion and glucose utilization, and impaired hepatic glucose metabolism, together with the activation of pro-inflammatory cytokines, culminating in T2DM [84]. It is well known that oxidative stress in pancreatic β-cells is induced by high glucose, hyperlipidemia, and inflammatory responses [85]. An established state of oxidative stress leads to increased production of proinflammatory cytokines, such as tumor necrosis factor (TNF) [86]. Given the above, probiotic therapies have been proposed as an alternative therapeutic strategy in the treatment and management of diabetes, specifically for key mediators of insulin resistance, such as TNF−α. Interestingly, a decrease in this proinflammatory marker was observed in two studies [77,78], whereas no changes were observed in three other studies [38,72,74]. It is noteworthy that in those studies where a change was observed, multistrain probiotics were used [77,78], which may suggest that they are more effective compared to mono-strain probiotics.

On the other hand, there is no clear trend between the intake of probiotics and the concentration of hs−CRP and interleukins. hs−CRP remained unchanged after eight weeks [72], twelve weeks [76], and six months [38] of monostrain probiotic intake. However, an increase in this biomarker was observed after four months of monostrain probiotic intake [74] and a reduction with a multistrain probiotic during six months [78]. Similar behavior was observed with interleukins, with multistrain probiotics being more effective.

Regarding the adipocytokines, adiponectin is the most abundant and is known to have a regulatory effect on glucose and lipid metabolism [87]. As shown in Table 2, in most studies there is no association between probiotic consumption and this biomarker. On the other hand, only one study using a multistrain probiotic and lasting six months showed an increase in adiponectin levels [78]. Although leptin, an adipokine whose primary function is to regulate energy balance, has been found to mediate insulin secretion and sensitivity in peripheral tissues [88], only one study evaluated this biomarker and found no change after probiotic supplementation [76].

Patients with T2DM often have comorbidities, including dyslipidemia and hypertension. Diabetes-related changes in plasma lipid levels are among the key factors that are amenable to intervention [89]. Based on the included articles, the lipid profile of patients with T2DM is modestly changed by probiotic supplementation. The most modifiable parameter was total cholesterol, which was reduced in four of the interventional studies. On the other hand, triglycerides and lipoprotein cholesterol (HDLc and LDLc) seemed to be more resistant to change with probiotic supplementation in T2DM patients. TG reduction was only observed in studies using multistrain probiotics [75,78]. On the other hand, no relationship was observed for HDLc and LDLc. Monostrain soy milk lowers LDL and raises HDL [72], and multistrain capsules raise HDL [79].

### 3.3. Metabolic Syndrome

MetS is a cluster of interrelated risk factors that increase the likelihood of developing CVDs, T2DM, and other health complications [90]. A recent study revealed that MetS and its related cardiometabolic components are highly prevalent worldwide [91]. According to ATP III classification, diagnosis of MetS is established if three out of the following five parameters are pathologically altered: waist circumference (>102 cm for men and >88 cm for women), blood pressure (>135/85 mm/Hg), fasting blood glucose (>6.1 mmol/L), triglyceride (>1.7 mmol/L), and HDLc (<1.03 mmol/L for men and <1.29 mmol/L for women) [92].

Lifestyle modifications, particularly dietary habits, are the main strategy for the prevention and management of MetS [93]. Changes in dietary habits can ameliorate obesity and insulin resistance, which play a key role in these pathological conditions [94]. While probiotics show potential, their effects on MetS markers remain insufficiently understood, limiting their application in the prevention and treatment of MetS in clinical practice [95]. From 2015 to date, the effect of probiotic yogurt [96,97], kefir [96,97,98,99,100], Yakult [101], milk [102,103], and capsules and/or sachets containing strains [16,104] has been studied in MetS patients (Table 3).

Based on the findings of the articles included in this review, probiotic consumption leads to only modest changes in the anthropometric characteristics of patients. Only one study found a significant decrease in BMI [102], and no changes in waist circumference were observed. Strain-specific responses could explain these minor changes because different probiotic strains may have variable effects on gut metabolism and microbiota, which could lead to heterogeneous results.

Regarding blood pressure, 50% of the studies found no significant changes following probiotic consumption [16,100,101,102,103]. Meanwhile, other studies observed a reduction in both SBP and DBP after three months of daily kefir intake [98,99].

With regard to glucose, three studies showed a clear decrease in its concentration after kefir [98], yogurt [96], and probiotic powder [105] intake (Table 3), while four studies did not show any relationship between glucose levels and probiotic intake [16,99,100,102]. On the other hand, probiotic intake was specifically associated with varying effects on insulin levels and insulin resistance, including reductions [99,105], increases [96], and no significant changes [16,100,102].

Furthermore, even though uric acid is known to correlate with MetS [106], this biomarker has only been measured in one study and was found to decrease after 2 months of daily yogurt intake [97].

Homocysteine has been recognized as a potential marker for atherosclerosis progression [107]. However, very few studies evaluate this marker after sustained probiotic intake. To our knowledge, only three studies followed up on this biomarker, and two of them showed a reduction in its levels [100,103].

Concerning the relationship between probiotic consumption and lipid profile (see Table 3), the circulating levels of both total cholesterol and triglycerides remain unchanged in most of the studies. A decrease in total cholesterol [102] and triglycerides [105] was observed. As for lipoprotein-cholesterol, a positive relationship between probiotic consumption and a reduction in LDLc was observed in three studies using kefir [98], probiotic yogurt [102], and probiotic powder [105], though in other studies the levels remained significantly unchanged [16,99,100,101]. However, HDLc levels appear to be less responsive to probiotic consumption in patients with MetS [16,98,99,100,101,102].

It is important to note that in the 12-week intervention study with kefir carried out by da Silva Ghizi et al. [98], differences in HDLc levels were observed only in women. Likewise, daily intake of Yakult for 12 weeks did not induce changes in VLDLc levels [101].

Many of the adipokines exert multiple actions in a variety of cellular processes, leading to a complex array of abnormal characteristics in MetS [108]. For this reason, adipokines are target markers for assessing the effects of probiotic consumption in MetS patients.

Regarding TNF–α, the daily intake of kefir [99,100] and probiotic milk [102] leads to its decrease. However, consumption of a probiotic freeze-dried *L. helveticus* R0052 and *B. longum* R0175 (CNCM strain I-3470) bacteria does not affect the content of this marker after two months [104], and *L. reuteri* V3401 does not do so after three months [16].

With respect to IL–6, as presented in Table 3, three studies reported a significant reduction in this biomarker following the daily intake of probiotic milk [102,103] and kefir [100]. However, no changes in plasma levels of IL–1β and IL–8 were observed in the evaluated interventional studies. In addition, a reduction in plasma levels of IL-10 was found only in one 12-week study [100]. Circulating leptin levels showed no change, whereas adiponectin levels tended to increase after a daily intake of 80 mL of probiotic milk over 45 days [103], though no changes were detected in another study [16].

Levels of hs-CRP remain unchanged, regardless of probiotic type, dose, time of intervention, and sex in patients with MetS. A similar trend was observed for ICAM-1, sVCAM-1, and sICAM-1 (Table 3).

Although there is evidence of a positive association between circulating levels of TMAO and MetS [109], only one study evaluated this biomarker, showing that probiotic intake did not produce changes in its levels after 12 weeks [101].

### 3.4. Hypercholesterolemia

Hypercholesterolemia is one of the most important risk factors of atherosclerosis. Hypercholesterolemia is mostly defined by levels of total cholesterol > 200 mg/dL and LDLc ≥ 130 mg/dL (≥3.4 mmol/L) and/or non-HDLc ≥ 160 mg/dL (≥4.1 mmol/L) [110,111]. Hypercholesterolemia is also characterized by elevated levels of triglycerides and/or low HDLc levels. Due to the benefits of LDLc lowering in cardiovascular risk reduction, the principal’s goals of CVD prevention for LDLc are <1.8 mmol/L (<70 mg/dL), <2.6 mmol/L (<100 mg/dL), and <3.0 mmol/L (<116 mg/dL) in high, moderate and low cardiovascular risk [110,112,113,114], respectively.

The first steps for achieving a cholesterol-lowering effect involve lifestyle and nutritional modifications. Thus, the use of supplements and functional foods for the treatment of dyslipidemias is rising as a good option in individuals considered to be at low cardiovascular risk who are in a stage prior to requiring drug treatment or even in patients treated with pharmacological drugs who have not reached guideline-recommended LDLc levels [115]. In this sense, probiotics have been raised as a non-pharmacological tool for the management of dyslipidemia [116,117].

To the best of our knowledge, only six clinical trials conducted from 2015 to date [118,119,120,121,122,123] have evaluated the efficacy of probiotics on subjects defined as hypercholesterolemic but without other CVD risk factors or clinical conditions (Table 4). In these clinical trials, the administered probiotic bacteria belonged to lactic acid species known for their cholesterol-lowering capacity [116], except for one study that used the probiotic bacteria *S. boulardii* [121].

The lipid profile was thoroughly analyzed in the six clinical trials. Daily intake of multistrain milk (*Lactobacillus* and *Bifidobacterium* strains) for ten weeks reduced total cholesterol by 8.1% [118] as did the consumption of *L. plantarum* ECGC 13110402 [119]. However, the other four probiotic bacteria showed no impact on plasmatic cholesterol levels [120,121,122,123]. Regarding LDLc levels, a critical target for reducing cardiovascular risk, probiotic intake appears to be an effective strategy. Significant reductions were reported in four clinical trials [118,119,120,122], with reductions reaching up to 10.4% when a mix of three acid lactic bacteria was administered [118]. However, *Streptococcus thermophilus* YIT 2001 and *S. boulardii* failed to reduce LDLc levels [121,123]. In contrast, HDLc and triglyceride levels proved more challenging to modify through probiotic intervention. Only the study that used *L. plantarum* ECGC 13110402 strain achieved an increase in HDLc levels [119]. Triglyceride levels, on the other hand, were elevated after probiotic intervention in the single clinical trial that used a probiotic multistrain [118] but reduced after administration of *L. plantarum* ECGC 13110402 [119]. Additionally, probiotic intake was associated with a reduction in the concentration of VLDL and intermediate density lipoprotein (IDLc) particles [121]. As shown in Table 4, probiotics generally show a greater effect on LDLc reduction than HDLc elevation due to more defined mechanisms like bile acid metabolism, cholesterol assimilation, and SCFA production. Bile salt hydrolase (BSH) activity reduces cholesterol absorption by deconjugating BAs and promoting their excretion [124]. A decrease in BAs triggers an increase in de novo BA synthesis from cholesterol to restore balance. The conversion of cholesterol into BAs represents a major pathway for reducing serum cholesterol levels. Certain probiotics can also directly assimilate cholesterol into their cell membranes, limiting intestinal absorption. Additionally, some strains can convert cholesterol into coprostanol, a compound that is poorly absorbed and readily excreted in feces [125,126]. In contrast to the LDLc, HDLc modulation relies more on anti-inflammatory pathways and metabolic signaling, which are less direct and strain dependent [127]. The variability in outcomes underscores the importance of strain specificity, individual health status, and baseline cholesterol levels in determining the lipid-modulating effects of probiotics.

It is well known that hypercholesterolemic patients exhibit elevated levels of inflammatory markers [128]. Probiotics reduce inflammatory markers by modulating gut microbiota, strengthening gut barrier integrity, and decreasing systemic endotoxins. They lower levels of pro-inflammatory cytokines (e.g., TNF-α, IL-6, IL-1β) while increasing anti-inflammatory cytokines (e.g., IL-10) [129]. However, the effects remain inconsistent and are closely related to the probiotic strain and the health or disease status of the individual. Thus, in the included clinical trials, probiotic intake did not affect IL-10, IL-6 [119,120], CRP [119,120,121], and Lp(a) [121] levels. Nonetheless, TNFα levels were reduced with prolonged intake of *L. paracasei* TISTR 2593 [120], though no reduction was observed with *L. plantarum* ECGC 13110402 [119]. Regarding oxidation markers, probiotic intake was associated with reductions in malondialdehyde (MDA) [120] and MDA-LDL levels [123], as well as an increase in resistance to LDL oxidation [118].

### 3.5. Secondary Prevention in Coronary Artery Disease

CAD, also termed as coronary heart disease or ischemic heart disease, is one of the major CVDs affecting the world’s population and has atherosclerosis as the primary underlying cause [130]. CAD is the leading single cause of mortality and disability-adjusted life years worldwide. Much of this burden falls on low- and middle-income countries, accounting for nearly 7 million deaths and 129 million DALYs annually [131,132]. Since 2015, numerous human studies have been conducted to evaluate the effects of probiotic consumption on inflammation, glucometabolic parameters, and lipid markers in patients with established CAD [133,134,135,136,137,138]. The characteristics of the studies as well as the main findings are summarized in Table 5.

Two studies have evidenced that SBP responds more readily than DBP, with reductions observed after six [136] and eight weeks [138] of probiotic consumption in patients with coronary angiography and end-stage renal disease, respectively. In contrast, other studies lasting six and sixteen weeks found no changes in blood pressure following daily probiotic intake [135,137].

Dyslipidemia, a predominant cause and modifiable risk factor for CAD, is particularly significant, with 56% of CAD related to abnormal cholesterol levels [139]. As shown in Table 5, only in one study in patients with end-stage renal disease reported a decrease in total cholesterol after two months of probiotic intervention with one capsule containing 1.9 × 10^9^ CFU of *L. rhamnosus* per day [138]. In contrast, six [136,137], eight [133,138], and sixteen [135] weeks of probiotic supplementation do not seem to be sufficient to improve LDLc levels. However, a longer intervention time (6 months) led to a significant reduction in LDLc [134]. A similar trend was observed for HDLc. No changes were observed after six [136,137] and eight [138] weeks, but an increase was observed when the probiotic intervention was prolonged in time to six months [134]. In the four studies assessing triglyceride levels, no changes were observed at six [136,137], eight [138], or sixteen weeks [135].

Although leptin is essential for the maintenance of homeostasis in the cardiovascular system, only two of the five analyzed studies included this marker. The results showed that after daily intake of 80 mL of a probiotic with *L. plantarum* 299v for six weeks, leptin levels significantly decreased in participants with CAD [136,137]. Regarding the interleukins (IL-1β, IL–6, IL–8 and IL–12), these markers appear to be more easily modified, as a decrease in their content was observed (Table 5). In contrast, the anti-inflammatory IL-10 showed no change in its concentration in clinical trials lasting two months [133,138]. The immunomodulatory effects of probiotics on pro-inflammatory and anti-inflammatory pathways have been widely studied, as extensively reviewed by Cristofori et al. [129]. However, whether probiotics primarily target pro-inflammatory cytokines while leaving anti-inflammatory pathways largely unaffected remains a topic of debate. Probiotics predominantly reduce pro-inflammatory cytokines (e.g., TNF-α, IL-6, IL-1β) by modulating immune pathways such as NF-κB and MAPK, thereby decreasing systemic inflammation. They also produce SCFAs and other metabolites that further suppress inflammatory responses. While some strains (*Lactobacillus reuteri*, *Bifidobacterium bifidum*) can upregulate anti-inflammatory cytokines like IL-10, evidence for this effect remains less consistent and highly strain-dependent.

## 4. Final Remarks/Conclusions

This review, for the first time, compiles existing evidence from human studies on the effects of probiotic intake in vulnerable populations, including individuals with hypertension, T2DM, MetS, and hypercholesterolemia, and in secondary prevention in CAD. With the existing evidence, it could be suggested that, in populations at risk of CVD, probiotics may serve as a potential intervention tool in the prevention of CVD. Improvements have been observed in anthropometric measures, inflammation, blood pressure, markers of glucose metabolism, lipid profiles, and endothelial function. Nevertheless, it is crucial to adopt a pragmatic approach, given the heterogeneity of findings. These divergences may be attributed to factors such as probiotic composition (mono- or multistrain), characteristics of the delivery matrix (food, capsules, and sachets), intervention duration, dosage regimen, and the baseline health profiles of participants.

Further research is warranted to comprehensively elucidate the precise mechanisms by which probiotics exert their beneficial effects in the context of CVD. In addition, the evaluation of other factors that have an impact on the effect of probiotics needs further investigation, such as: (i) determining the optimal probiotic strain or strains, (ii) appropriate dosage, (iii) treatment duration, and (iv) optimal delivery vehicle. Moreover, exploring sex-specific differences is crucial, as current evidence in populations with cardiovascular risk factors remains limited in this area.

## Figures and Tables

**Table 1:** Effect of probiotics supplementation on inflammation markers in patients with hypertension.

Probiotic Strain (Product)	Dose (Intervention Time)	Study Characteristics (*n*)	Parameters Changing	Unchanging Parameters	Ref.
*L. casei* 01 (Cheese)	Non-specified (4 weeks)	Randomized double-blind pilot trial: Probiotic cheese (15) and conventional cheese (15)	↓ SBP and DBP ↓ TC and TG ↑ HDLc	LDLc	[61]
(1) *L. paracasei* LPC-37 (2) *L. rhamnosus* N001 (3) *L. acidophilus NCFM* (4) *B.lactis* HN019 (Sachet)	(1) 10^9^ CFU/day (2) 10^9^ CFU/day (3) 10^9^ CFU/day (4) 10^9^ CFU/day (8 weeks)	Randomized, triple-blind, placebo-controlled trial: Probiotic (19) and placebo (17)	↓ FBG ↓ TC ↑ HDLc	SBP, DBP, TG, VLDLc, LDLc, and hs–CRP,	[62]
*L. plantarum* DSM 5313 (Sachet)	1 × 10^9^ CFU/day (3 months)	Double-blind, placebo-RCT: Probiotic (44) and placebo (46)		SBP and DBP	[63]
(1) *L.acidophilus* LA-5 (2) B. *Bifidobacterium* B-12 (3) *S.thermophilus* STY-31 (4) *L. bulgaricus* LBY-27 (Capsules)	>4 × 10^9^ CFU/day (8 weeks)	Double-blind, placebo-RCT: Probiotic (32) and placebo (32)	↓ SBP ↓ DBP		[60]
(1) L. *acidophilus* LA-5 (2) B. *lactis* BB-12 (Yogurt and/or capsules)	3 × 10^9^ CFU/day or 6 × 10^9^ CFU/day (6 weeks)	Double-blinded, factorial, parallel study: Yogurt + ProC (40), Yogurt + PlaC (37), CM + ProC (39) and CM + PlaC (40)		SBP, DBP, TC, TG, HDLc and LDLc	[64]

CM: control milk; DBP: diastolic blood pressure; FBG: fasting blood glucose; HDLc: high-density lipoprotein cholesterol; PlaC: placebo capsules; ProC: probiotic capsules; RCT: randomized clinical trial; SBP: systolic blood pressure; TC: total cholesterol; TG: triglycerides; VLDLc: very low-density lipoprotein cholesterol. Downward arrows indicate decreased values. Upward arrows indicate increased values.

**Table 2:** Effect of probiotics supplementation on metabolic endotoxemia and inflammation markers in patients with T2DM.

Probiotic Strain (Product)	Dose (Intervention Time)	Study Characteristics (*n*)	Parameters Changing	Unchanging Parameters	Ref.
	4 × 10^9^ CFU/day (8 weeks)	Parallel, RCT: Probiotic (24), Control (24)		MDA	[71]
*L. plantarum* A7 (Soy milk)		Parallel, RCT: Probiotic (20) and control (20)	↓ LDLc ↑ HDLc	FBG, TNF−α, hs−CRP, adiponectin and TG	[72]
		Parallel, RCT: Probiotic (24) and control (24)	↓ Weight ↓ WHR ↓ BMI		[73]
(1) *B. bifidum* W23 (2) *B. lactis* W52 (3) *L. acidophilus* W37 (4) *L. brevis* W63 (5) *L. casei* W56 (6) *L. salivarius* W24 (7) *L. lactis* W19 (8) *L. lactis* W58 (Sachets)	10^10^ CFU/day (12 weeks)	Single-center, double-blind, placebo-RCT: Probiotic (48) and placebo (46)	↓ FBG, insulin, ↓ C−peptide ↓ HOMA−IR ↓ SBP, DBP ↓ TG, TC, LDLc	Weight, BMI, WHR, HDLc and TC/HDLc	[75]
*L. casei* (Fermented milk)	4 × 10^10^ CFU/day (16 weeks)	RCT: Probiotic (34) and control (34)	↑ HbA1c ↑ hs−CRP	BMI, FBG, C−peptide, TNF−α, IL−6, adiponectin, TC, TG and HDLc	[74]
*L. reuteri* DSM 17938 (Tablets)	10^8^ CFU/day or 10^10^ CFU/day (12 weeks)	Double-blind, placebo-RCT: Placebo (>12), low (>12) and high (>12)	↑ Weight (low group) ↑ BMI (low group)	WC, FBG, insulin, HbA1c, hs−CRP, adiponectin, leptin, SBP, DPB, TC, TG, HDLc, LDLc	[76]
*L. reuteri* (Capsules)	ADR-1: 4 × 10^9^ CFU/day ADR-3: 2 × 10^10^ cells/day (6 months)	Double-blind, placebo-RCT: ADR-1 (22), ADR-3 (24) and placebo (24)	↓ HbA1c ↓ TC ↓ SBP	FBG, insulin, HOMA−IR, C−peptide, hs−CRP, IL−6, IL−10, IL−17, TNF−α, IL−1β, LDLc, HDLc, TG, DBP	[38]
Concentrated of 14 probiotic bacteria genera (1) *Bifidobacterium* (2) *Lactobacillus* (3) *Lactococcus* (4) *Propionibacterium* (5) *Acetobacter* (Sachets)	(1) 10^11^ CFU/day (2) (3) 6 × 10^11^ CFU/day (4) 3 × 10^11^ CFU/day (5) 10^7^ CFU/day (8 weeks)	Single-center double blind, placebo, parallel, RCT: Probiotic (31) and placebo (22)	↓ Weight, WC, BMI ↓ HOMA−IR ↓ TNF−α ↓ IL−6, IL−8, IL−17, IL−1β	FBG, insulin, HbA1c	[77]
(1) *B. bifidum* W23 (2) *B. lactis* W52 (3) *L. acidophilus* W37 (4) *L. brevis* W63 (5) *L. casei* W56 (6) *L. salivarius* W24 (7) *L.lactis* W19 (8) *L. lactis* W58 (Sachets)	10^10^ CFU (6 months)	Double-blind, placebo-RCT: Probiotic (31) and placebo (30)	↓ FBG ↓ Insulin ↓ HOMA−IR ↓ hs−CRP, ↓ TNF−α, IL−6 ↑ Adiponectin ↓ TG and TC	BMI, WHR, C-peptide, SBP, DBP	[78]
(1) *L.acidophilus* (2) *L.casei* (3) *L. rhamnosus* (4) *L. bulgaricus* (5) *B. breve* (6) *B. longum* (7) *S. thermophilus* (Capsules)	(1) 4 × 10^9^ CFU/day (2) 14 × 10^9^ CFU/day (3) 3 × 10^9^ CFU/day (4) 4 × 10^8^ CFU/day (5) 6 × 10^10^ CFU/day (6) 14 × 10^9^ CFU/day (7) 3 × 10^9^ CFU/day (6 weeks)	Double-blind, placebo-RCT: Probiotic (30) and placebo (30)	↓ FBG ↑ HDLc	Weight, WC, BMI, insulin, HOMA−IR, TC, TG and LDLc	[79]
*L. casei* (Capsules)	1 × 10^8^ CFU/day (6 weeks)	Parallel, RCT: Probiotic (20) and placebo (20)	↓ FBG ↓ Insulin ↓ HOMA−IR ↓ TC	HbA1c	[80]
(1) *L. salivarius* BLS22 (2) *L. casei* UBLC42 (3) *L. plantarum* BLP40 (4) *L. acidophilus* UBLA34 (5) *B. breve* UBBr01 (6) *B. coagulans* Unique IS2 (Capsules)	6 × 10^10^ CFU/day (12 weeks)	Parallel, RCT: Probiotic (40) and placebo (40)	↓ FBG ↓ HbAIc	Weight, insulin, HOMA−IR, TC, TG, LDLc and HDLc	[81]
(1) *L.acidophilus* (2) *L.plantarum* (3) *L.fermentum* (4) *L.Gasseri* (Capsules)	(1) 5 × 10^10^ CFU/day (2) 1.5 × 10^10^ CFU/day (3) 7 × 10^9^ CFU/day (4) 2 × 10^10^ CFU/day (8 weeks)	Double-blind, placebo-RCT: Probiotic (34) and placebo (34)	↓ SBP ↓ DBP	TC/HDLc and LDLc/HDLc	[82]

BMI: body mass index; DBP: diastolic blood pressure; FBG: fasting blood glucose; HDLc: high-density lipoprotein cholesterol; HbA1c: glycated hemoglobin; hs-CRP: high-sensitivity C-reactive protein; HOMA-IR: homeostatic model assessment of insulin resistance; IL: interleukin; LDLc: low-density lipoprotein cholesterol; MDA: malondialdehyde; TC: total cholesterol; TG: triglycerides; SBP: systolic blood pressure; TNF−α: tumor necrosis factor−α; WC: waist circumference; WHR: waist-hip ratio. Downward arrows indicate decreased values. Upward arrows indicate increased values.

**Table 3:** Effect of probiotics supplementation on metabolic endotoxemia and inflammation markers in patients with metabolic syndrome.

Probiotic Strain (Product)	Dose (Intervention Time)	Study Characteristics (*n*)	Parameters Changing	Unchanging Parameters	Ref.
(1) *L. lactis* ssp. *lactis* (2) *L. lactis* ssp. *cremoris* (3) *L. lactis* ssp. *diacetylactis*, (4) *L. mesenteroides* ssp. *cremoris* (5) *L. kefir* (6) *K.marxianus* (7) *S. unisporus* (Kefir)	180 mL of kefir/day (CFU non-specified) (12 weeks)	Parallel-group, RCT: Kefir (12) and control (10)	↓ Insulin ↓ HOMA–IR ↓ TNF–α ↓ SBP and DBP	BMI, WC, TC, HDLc, LDLc, TG, homocysteine, hs–CRP, IL–6, IL–10, glucose	[99]
Non-specified (Kefir)	Men: 1.6 mL of kefir/kg body weight/day Women: 1.9 mL of kefir/kg body weight/day (CFU non-specified) (12 weeks)	Double-blind placebo-RCT: Kefir (24) and control (24)	↓ SBP and DBP ↓ FBG ↓ LDLc ↓ Non-HDLc, ↓ TG ↓ oxLDL ↑ HDLc (Women)	TC, HDLc (men) and hs-CRP	[98]
(1) *L. bulgaricus* (2) *S. thermophilus* (3) *B. lactis* Bb12 (4) *L. acidophilus* La5 (Yakult)	First Day of Production: (3) 1.5 × 10^9^ CFU/day (4)1.9 × 10^9^ CFU/day (2 months)	Double-blind placebo-RCT: Yogurt (22) and control (22)	↓ FBG, CAM-1 ↑ Insulin ↑ HOMA–IR	HOMA-β and ICAM-1	[96]
*L. casei* Shirota (Milk)	1.95 × 10^10^ CFU/day (12 weeks)	Single-center, prospective, RCT: Probiotic (13) and Control (15)		SBP, DBP, TC, TG, HDLc, LDLc, VLDLc, hs-CRP, sVCAM-1, sICAM-1 and TMAO	[101]
*B. lactis* HN019 (Milk)	2.72 × 10^10^ CFU/day (45 days)	Randomized Trial: Probiotic (26) and control (25)	↓ BMI ↓ TC and LDLc ↓ TNF–α and IL–6	WC, SBP, DBP, TG, HDLc, glucose, insulin and HOMA–IR	[102]
	2.72 × 10^10^ CFU/day (90 days)	Double-blind placebo-RCT: NoMetS (14) and MetS (19)	↓ IL–6 ↓ Homocysteine ↑ Adiponectin ↑ NO	WC, SBP, DBP, hs-CRP and leptin	[103]
(1) *L. bulgaricus* (2) *S. thermophilus* (3) *B. lactis* Bb12 (4) *L. acidophilus* La5 (Yogurt)	First Day of Production: (3) 1.5×10^9^ CFU/day (4) 1.9×10^9^ CFU/day (8 weeks)	Double-blind placebo-RCT: Probiotic (22) and control (22)	↓ Uric acid	MDA and oxLDL	[97]
*L. reuteri* V3401 (Capsule)	5 × 10^9^ CFU/day (12 weeks)	Randomized, crossover, placebo-controlled, single-center trial: Group 1 (28) and group 2 (25)		BMI, SBP, DBP, glucose, insulin, HOMA–IR, TC, TG, LDLc, HDLc, hs-CRP, IL–6, IL-8, TNF–α, adiponectin, leptin and sVCAM-1	[16]
(1) *L. helveticus R0052* (2) *B. longum R0175* (Sachets)	1 × 10^10^ CFU/day (8 weeks)	Double-blind, placebo-RCT: Probiotic (27) and placebo (22)		TNF–α, IL–1β, IL–6 and IL–10	[104]
(1) *L. lactis* subsp. *lactis* (2) *L. lactis* subsp. *cremoris* (3) *Ls. lactis* subsp. *diacetylactis* (4) *L. mesenteroides* subsp. *cremoris* (5) *L. kefyr* (6) *K. marxianus* (7) *S.unisporus* (Kefir)	(1), (2), (3), (4), (5) Minimum 10⁶ UFC/g/day (6), (7) Minimum 10^5^ UFC/g/day (12 weeks)	Randomized controlled clinical trial: Kefir (31) and milk (31)	↓ Homocysteine ↓ TNF–α ↓ IL–6 ↓ IL-10	BMI, WC. SBP, DBP, glucose, insulin, HOMA–IR, TC, TG, HDLc, LDLc, ApoA1, ApoB, Lp(a), hs-CRP	[100]
*Bifidobacterium adolescentis* CCFM8630 and *Lactobacillus reuteri* CCFM8631	1 × 10^10^ CFU (11 weks)	Randomized, placebo-controlled clinical trial: Probiotic (21) and placebo (19)	↓ FBG ↓ Insuline ↓ TG ↓ LDLc	**TC**	[105]

BMI: body mass index; DBP: diastolic blood pressure; FBG: fasting blood glucose; HDLc: high-density lipoprotein cholesterol; HbA1c: glycated hemoglobin; hs-CRP: high-sensitivity C-reactive protein; HOMA-IR: homeostatic model assessment of insulin resistance; ICAM, intercellular adhesion molecule cell; IL: interleukin; LDLc: low-density lipoprotein cholesterol; MDA: malondialdehyde; TC: total cholesterol; TG: triglycerides; sICAM-1: soluble intercellular adhesion molecule 1; SBP: systolic blood pressure; sVCAM-1: soluble vascular cell adhesion molecule 1; TNF−α: tumor necrosis factor−α; VCAM: vascular cell adhesion molecule cell; WC: waist circumference; WHR: waist-hip ratio. Downward arrows indicate decreased values. Upward arrows indicate increased values.

**Table 4:** Studies evaluating the effect of probiotic supplementation in hypercholesterolemic patients.

Probiotic Strain (Product)	Dose (Intervention Time)	Study Characteristics (*n*)	Parameters Changing	Unchanging Parameters	Ref.
(1) *L. acidophilus* La5 (2) *L. casei* TMC (3) *B. lactis* Bb12 (Skimmed milk drink)	(1) 1.2 × 10^8^ CFU/day (2) 1.2 × 10^8^ CFU/day (3) 1.2 × 10^8^ CFU/day (10 weeks)	Double-blind, placebo-RCT: Probiotic (20) and placebo (20)	↑ LDL-OXI lag time ↓ TC and LDLc	BMI, body weight, HDLc	[118]
*L. plantarum* ECGC 13110402 (Capsules)	4 × 10^9^ CFU/day (12 weeks)	Single-center, prospective, randomized, placebo- controlled, parallel-group: Probiotic (23) and placebo (23)	↓ LDLc ↓ TC ↓ TG ↑ HDLc ↓ SBP	Body weight, BMI, waist, DBP, IL-6, TNF-α, CRP and IL-10.	[119]
*L. Paracasei* TISTR 2593 (Capsules)	3.675 × 10^9^ CFU/day (90 days)	Single-center, prospective, randomized, double-blind, placebo-controlled, parallel-group trial: Probiotic (21) and placebo (21)	↓ LDLc ↓ MDA ↓ TNF-α ↑ Adiponectin ↑ ApoE	BMI, SBP, DBP, FBG, TC, TG, HDLc, IL-10, IL-6	[120]
*S. boulardii* (Capsules)	1.12 × 10^11^ CFU/day (8 weeks)	Single-arm, open-label pilot study: (11)	↓ VLDLp ↓ ILDp	BMI, TC, LDLc, HDLc, TG, VLDLp, LDLp, HDLp, non-HDLp, ApoB-100, Lp(a), hs-CRP, insulin, homocysteine	[121]
*L. reuteri* (Capsules)	Week 1 and 2: 3 × 10^9^ CFU/day Week 3: 6 × 10^9^ CFU/day Week 4: 9 × 10^9^ CFU/day	Pilot, randomized, dose-escalation design: Standard capsules-SC (5) and delayed release capsules-DC (5)	↓ LDLc with DC	TC, HDLc, TG	[122]
*S. thermophilus* YIT 2001 (Fermented milk)	1 × 10^11^ CFU/day (12 weeks)	Randomized, double-blind, placebo-controlled: Probiotic (29) and placebo (30)	↓ MDA-LDL, ↓ MDA-LDL/LDLc ↓ SBP and DBP	TC, LDLc, HDLc and TG	[123]

Apo: apolipoprotein; BMI: body mass index; DBP: diastolic blood pressure; FBG: fasting blood glucose; HDLc: high-density lipoprotein cholesterol; HbA1c: glycated hemoglobin; hs-CRP: high-sensitivity C-reactive protein; HOMA-IR: homeostatic model assessment of insulin resistance; ICAM, intercellular adhesion molecule cell; IL: interleukin; LDLc: low-density lipoprotein cholesterol; MDA: malondialdehyde; OXI: oxidation; P: particles. TC: total cholesterol; TG: triglycerides; sICAM-1: soluble intercellular adhesion molecule 1; SBP: systolic blood pressure; sVCAM-1: soluble vascular cell adhesion molecule 1; TNF−α: tumor necrosis factor−α; VCAM: vascular cell adhesion molecule cell; WC: waist circumference; WHR: waist-hip ratio. Downward arrows indicate decreased values. Upward arrows indicate increased values.

**Table 5:** Studies evaluating the effect of probiotic supplementation in CAD patients.

Probiotic Strain (Product)	Dose (Intervention Time)	Study Characteristics (*n*)	Parameters Changing	Unchanging Parameters	Ref.
*L. rhamnosus* (Capsules)	1.6 × 10^9^ CFU/day (12 weeks)	Double-blind, placebo-RCT: LGG (22), Placebo (22)	↓ IL–1β ↓ LPS	IL–10	[133]
*L. rhamnosus* (Capsules)	1.6 × 10^9^ CFU/day (2 months)	Double-blind, placebo-RCT in end-stage renal disease patients: Probiotic (24), Placebo (24)	↓ SBP ↓ TC ↓ LPS ↓ TNF–α	DBP, LDLc, HDLc, TG, IL–10 and hs–CRP	[138]
*L. rhamnosus* (Sachet)	3 × 10^10^ CFU/day (6 months)	Double-blind, placebo-RCT: Probiotic (36), Placebo (24)	↓ IL–6 ↓ LDLc	Creatinine	[134]
(1) *B. bifidum* (2) *L. casei* (3) *L. acidophilus* (Capsules)	(1), (2), (3) 2 × 10^9^ CFU/day (12 weeks)	Double-blind, placebo-RCT in patients with 2- and 3-vessel CHD: Probiotic (30), Placebo (30)	↓ FBG, insulin ↓ HOMA-IR ↓ TC/HDLc ratio ↓ hs–CRP ↓ NO ↑ HDLc	VLDLc, LDLc, TC, TG, SBP and DBP	[135]
*L. plantarum* 299v (Juice drink)	2 × 10^10^ CFU/day (6 weeks)	Non-randomized intervention in coronary angiography patients: Lp299v (20)	↓ SBP ↓ IL–8 and IL–12 ↓ Leptin	DBP, TC, TG, LDLc and HDLc	[136]
*L. plantarum* 299v (Juice drink)	2 × 10^10^ CFU/day (6 weeks)	Non-randomized intervention in coronary angiography patients: Lp299v (15)	↓ Leptin ↓ IL–8 and IL–12	DBP, SBP, FBG, TC, TG, HDLc and LDLc	[137]

BMI: body mass index; DBP: diastolic blood pressure; FBG: fasting blood glucose; HDLc: high-density lipoprotein cholesterol; HbA1c: glycated hemoglobin; hs-CRP: high-sensitivity C-reactive protein; HOMA-IR: homeostatic model assessment of insulin resistance; IL: interleukin; LDLc: low-density lipoprotein cholesterol; LPS: lipopolysaccharide; MDA: malondialdehyde, NO: nitric oxide; TC: total cholesterol; TG: triglycerides; SBP: systolic blood pressure; TNF−α: tumor necrosis factor−α; WC: waist circumference; WHR: waist-hip ratio. Downward arrows indicate decreased values. Upward arrows indicate increased values.

## References

[1] Metchnikoff E., Metchnikoff I.I. (1908). The Prolongation of Life: Optimistic Studies.

[2] World Health Organization, Food and Agricultural Organization of the United Nations (2001). Health and Nutritional Properties of Probiotics in Food Including Powder Milk with Live Lactic Acid Bacteria.

[3] Hill C., Guarner F., Reid G., Gibson G.R., Merenstein D.J., Pot B., Morelli L., Canani R.B., Flint H.J., Salminen S. (2014). The International Scientific Association for Probiotics and Prebiotics Consensus Statement on the Scope and Appropriate Use of the Term Probiotic. Nat. Rev. Gastroenterol. Hepatol..

[4] George Kerry R., Patra J.K., Gouda S., Park Y., Shin H.S., Das G. (2018). Benefaction of Probiotics for Human Health: A Review. J. Food Drug Anal..

[5] Thushara R.M., Gangadaran S., Solati Z., Moghadasian M.H. (2016). Cardiovascular Benefits of Probiotics: A Review of Experimental and Clinical Studies. Food Funct..

[6] Timmis A., Townsend N., Gale C.P., Torbica A., Lettino M., Petersen S.E., Mossialos E.A., Maggioni A.P., Kazakiewicz D., May H.T. (2020). European Society of Cardiology: Cardiovascular Disease Statistics 2019. Eur. Heart J..

[7] Chakaroun R.M., Olsson L.M., Bäckhed F. (2022). The Potential of Tailoring the Gut Microbiome to Prevent and Treat Cardiometabolic Disease. Nat. Rev. Cardiol..

[8] Rittiphairoj T., Pongpirul K., Janchot K., Mueller N.T., Li T. (2021). Probiotics Contribute to Glycemic Control in Patients with Type 2 Diabetes Mellitus: A Systematic Review and Meta-Analysis. Adv. Nutr..

[9] Song E.J., Han K., Lim T.J., Lim S., Chung M.J., Nam M.H., Kim H., Nam Y. (2020). Do Effect of Probiotics on Obesity-Related Markers per Enterotype: A Double-Blind, Placebo-Controlled, Randomized Clinical Trial. EPMA J..

[10] Padro T., Santisteban V., Huedo P., Puntes M., Aguiló M., Espadaler-Mazo J., Badimon L. (2024). *Lactiplantibacillus plantarum* Strains KABP011, KABP012, and KABP013 Modulate Bile Acids and Cholesterol Metabolism in Humans. Cardiovasc. Res..

[11] Ravera A., Carubelli V., Sciatti E., Bonadei I., Gorga E., Cani D., Vizzardi E., Metra M., Lombardi C. (2016). Nutrition and Cardiovascular Disease: Finding the Perfect Recipe for Cardiovascular Health. Nutrients.

[12] Oniszczuk A., Oniszczuk T., Gancarz M. (2021). Role of Gut Microbiota, Probiotics and Prebiotics in the Cardiovascular Diseases. Molecules.

[13] Bagarolli R.A., Tobar N., Oliveira A.G., Araújo T.G., Carvalho B.M., Rocha G.Z., Vecina J.F., Calisto K., Guadagnini D., Prada P.O. (2017). Probiotics Modulate Gut Microbiota and Improve Insulin Sensitivity in DIO Mice. J. Nutr. Biochem..

[14] Plaza-Díaz J., Fernández-Caballero J.Á., Chueca N., García F., Gómez-Llorente C., Sáez-Lara M.J., Fontana L., Gil Á. (2015). Pyrosequencing Analysis Reveals Changes in Intestinal Microbiota of Healthy Adults Who Received a Daily Dose of Immunomodulatory Probiotic Strains. Nutrients.

[15] Toscano M., De Grandi R., Miniello V.L., Mattina R., Drago L. (2017). Ability of Lactobacillus Kefiri LKF01 (DSM32079) to Colonize the Intestinal Environment and Modify the Gut Microbiota Composition of Healthy Individuals. Dig. Liver Dis..

[16] Tenorio-Jiménez C., Martínez-Ramírez M.J., Del Castillo-Codes I., Arraiza-Irigoyen C., Tercero-Lozano M., Camacho J., Chueca N., García F., Olza J., Plaza-Díaz J. (2019). *Lactobacillus reuteri* V3401 Reduces Inflammatory Biomarkers and Modifies the Gastrointestinal Microbiome in Adults with Metabolic Syndrome: The PROSIR Study. Nutrients.

[17] Wang C., Nagata S., Asahara T., Yuki N., Matsuda K., Tsuji H., Takahashi T., Nomoto K., Yamashiro Y. (2015). Intestinal Microbiota Profiles of Healthy Pre-School and School-Age Children and Effects of Probiotic Supplementation. Ann. Nutr. Metab..

[18] Badimon L., Chagas P., Chiva-Blanch G. (2017). Diet and Cardiovascular Disease: Effects of Foods and Nutrients in Classical and Emerging Cardiovascular Risk Factors. Curr. Med. Chem..

[19] Hendijani F., Akbari V. (2018). Probiotic Supplementation for Management of Cardiovascular Risk Factors in Adults with Type II Diabetes: A Systematic Review and Meta-Analysis. Clin. Nutr..

[20] Dixon A., Robertson K., Yung A., Que M., Randall H., Wellalagodage D., Cox T., Robertson D., Chi C., Sun J. (2020). Efficacy of Probiotics in Patients of Cardiovascular Disease Risk: A Systematic Review and Meta-Analysis. Curr. Hypertens. Rep..

[21] Sun J., Buys N. (2015). Effects of Probiotics Consumption on Lowering Lipids and CVD Risk Factors: A Systematic Review and Meta-Analysis of Randomized Controlled Trials. Ann. Med..

[22] Olas B. (2020). Probiotics, Prebiotics and Synbiotics—A Promising Strategy in Prevention and Treatment of Cardiovascular Diseases?. Int. J. Mol. Sci..

[23] Ghanbari F., Hasani S., Aghili Z.S., Asgary S. (2024). The Potential Preventive Effect of Probiotics, Prebiotics, and Synbiotics on Cardiovascular Risk Factors through Modulation of Gut Microbiota: A Review. Food Sci. Nutr..

[24] Derrien M., van Hylckama Vlieg J.E.T. (2015). Fate, Activity, and Impact of Ingested Bacteria within the Human Gut Microbiota. Trends Microbiol..

[25] Plaza-Diaz J., Ruiz-Ojeda F.J., Gil-Campos M., Gil A. (2019). Mechanisms of Action of Probiotics. Adv. Nutr..

[26] Alp D., Kuleaşan H. (2019). Adhesion Mechanisms of Lactic Acid Bacteria: Conventional and Novel Approaches for Testing. World J. Microbiol. Biotechnol..

[27] Flach J., van der Waal M.B., van den Nieuwboer M., Claassen E., Larsen O.F.A. (2018). The Underexposed Role of Food Matrices in Probiotic Products: Reviewing the Relationship between Carrier Matrices and Product Parameters. Crit. Rev. Food Sci. Nutr..

[28] Ranadheera R.D.C.S., Baines S.K., Adams M.C. (2010). Importance of Food in Probiotic Efficacy. Food Res. Int..

[29] Timmerman H.M., Koning C.J.M., Mulder L., Rombouts F.M., Beynen A.C. (2004). Monostrain, Multistrain and Multispecies Probiotics—A Comparison of Functionality and Efficacy. Int. J. Food Microbiol..

[30] Wu L., Sun D. (2017). Consumption of Yogurt and the Incident Risk of Cardiovascular Disease: A Meta-Analysis of Nine Cohort Studies. Nutrients.

[31] Alard J., Lehrter V., Rhimi M., Mangin I., Peucelle V., Abraham A.L., Mariadassou M., Maguin E., Waligora-Dupriet A.J., Pot B. (2016). Beneficial Metabolic Effects of Selected Probiotics on Diet-Induced Obesity and Insulin Resistance in Mice Are Associated with Improvement of Dysbiotic Gut Microbiota. Environ. Microbiol..

[32] Kazemi A., Soltani S., Ghorabi S., Keshtkar A., Daneshzad E., Nasri F., Mazloomi S.M. (2020). Effect of Probiotic and Synbiotic Supplementation on Inflammatory Markers in Health and Disease Status: A Systematic Review and Meta-Analysis of Clinical Trials. Clin. Nutr..

[33] Government of Canada Natural Health Products Ingredients Database. https://webprod.hc-sc.gc.ca/nhpid-bdipsn/.

[34] Ministero della Salute (2018). Guidelines on Probiotics and Prebiotics.

[35] Morelli L., Pellegrino P. (2021). A Critical Evaluation of the Factors Affecting the Survival and Persistence of Beneficial Bacteria in Healthy Adults. Benef. Microbes.

[36] Szulińska M., Łoniewski I., Skrypnik K., Sobieska M., Korybalska K., Suliburska J., Bogdański P. (2018). Multispecies Probiotic Supplementation Favorably Affects Vascular Function and Reduces Arterial Stiffness in Obese Postmenopausal Women—A 12-Week Placebo-Controlled and Randomized Clinical Study. Nutrients.

[37] Qi D., Nie X.L., Zhang J.J. (2020). The Effect of Probiotics Supplementation on Blood Pressure: A Systemic Review and Meta-Analysis. Lipids Health Dis..

[38] Hsieh M.C., Tsai W.H., Jheng Y.P., Su S.L., Wang S.Y., Lin C.C., Chen Y.H., Chang W.W. (2018). The Beneficial Effects of *Lactobacillus reuteri* ADR-1 or ADR-3 Consumption on Type 2 Diabetes Mellitus: A Randomized, Double-Blinded, Placebo-Controlled Trial. Sci. Rep..

[39] Ranadheera C.S., Vidanarachchi J.K., Rocha R.S., Cruz A.G., Ajlouni S. (2017). Probiotic Delivery through Fermentation: Dairy vs. Non-Dairy Beverages. Fermentation.

[40] Damián M.R., Cortes-Perez N.G., Quintana E.T., Ortiz-Moreno A., Noguez C.G., Cruceño-Casarrubias C.E., Pardo M.E.S., Bermúdez-Humarán L.G. (2022). Functional Foods, Nutraceuticals and Probiotics: A Focus on Human Health. Microorganisms.

[41] Companys J., Pla-Pagà L., Calderen-Pérez L., Llaurade E., Solà R., Pedret A., Valls R.M. (2020). Fermented Dairy Products, Probiotic Supplementation, and Cardiometabolic Diseases: A Systematic Review and Meta-Analysis. Adv. Nutr..

[42] Kazemian N., Mahmoudi M., Halperin F., Wu J.C., Pakpour S. (2020). Gut Microbiota and Cardiovascular Disease: Opportunities and Challenges. Microbiome.

[43] Appelman Y., van Rijn B.B., ten Haaf M.E., Boersma E., Peters S.A.E. (2014). Sex Differences in Cardiovascular Risk Factors and Disease Prevention. Atherosclerosis.

[44] Fuchs F.D., Whelton P.K. (2020). High Blood Pressure and Cardiovascular Disease. Hypertension.

[45] Vasquez E.C., Pereira T.M.C., Peotta V.A., Baldo M.P., Campos-Toimil M. (2019). Probiotics as Beneficial Dietary Supplements to Prevent and Treat Cardiovascular Diseases: Uncovering Their Impact on Oxidative Stress. Oxid. Med. Cell. Longev..

[46] Pelton R. (2020). Postbiotic Metabolites: How Probiotics Regulate Health. Integr. Med. A Clin. J..

[47] Du Y., He C., An Y., Huang Y., Zhang H., Fu W., Wang M., Shan Z., Xie J., Yang Y. (2024). The Role of Short Chain Fatty Acids in Inflammation and Body Health. Int. J. Mol. Sci..

[48] Vourakis M., Mayer G., Rousseau G. (2021). The Role of Gut Microbiota on Cholesterol Metabolism in Atherosclerosis. Int. J. Mol. Sci..

[49] Upadrasta A., Madempudi R.S. (2016). Probiotics and Blood Pressure: Current Insights. Integr. Blood Press. Control.

[50] FitzGerald R.J., Murray B.A., Walsh D.J. (2004). Hypotensive Peptides from Milk Proteins. J. Nutr..

[51] Daliri E.B.M., Lee B.H., Oh D.H. (2017). Current Perspectives on Antihypertensive Probiotics. Probiotics Antimicrob. Proteins.

[52] Adams C., Sawh F., Green-Johnson J.M., Taggart H.J., Strap J.L. (2020). Characterization of Casein-Derived Peptide Bioactivity: Differential Effects on Angiotensin-Converting Enzyme Inhibition and Cytokine and Nitric Oxide Production. J. Dairy Sci..

[53] Gómez-Guzmán M., Toral M., Romero M., Jiménez R., Galindo P., Sanchez M., Zarzuelo M.J., Olivares M., Gálvez J., Duarte J. (2015). Antihypertensive Effects of Probiotics *Lactobacillus* Strains in Spontaneously Hypertensive Rats. Mol. Nutr. Food Res..

[54] Friques A.G.F., Arpini C.M., Kalil I.C., Gava A.L., Leal M.A., Porto M.L., Nogueira B.V., Dias A.T., Andrade T.U., Pereira T.M.C. (2015). Chronic Administration of the Probiotic Kefir Improves the Endothelial Function in Spontaneously Hypertensive Rats. J. Transl. Med..

[55] Kim D.Y., Park J.Y., Gee H.Y. (2023). *Lactobacillus plantarum* Ameliorates NASH-Related Inflammation by Upregulating L-Arginine Production. Exp. Mol. Med..

[56] Lau E., Neves J.S., Ferreira-Magalhães M., Carvalho D., Freitas P. (2019). Probiotic Ingestion, Obesity, and Metabolic-Related Disorders: Results from NHANES, 1999–2014. Nutrients.

[57] Lewis-Mikhael A.M., Davoodvandi A., Jafarnejad S. (2020). Effect of Lactobacillusplantarum Containing Probiotics on Blood Pressure: A Systematic Review and Meta-Analysis. Pharmacol. Res..

[58] Chi C., Li C., Wu D., Buys N., Wang W., Fan H., Sun J. (2020). Effects of Probiotics on Patients with Hypertension: A Systematic Review and Meta-Analysis. Curr. Hypertens. Rep..

[59] Ejtahed H.S., Ardeshirlarijani E., Tabatabaei-Malazy O., Hoseini-Tavassol Z., Hasani-Ranjbar S., Soroush A.R., Larijani B. (2020). Effect of Probiotic Foods and Supplements on Blood Pressure: A Systematic Review of Meta-Analyses Studies of Controlled Trials. J. Diabetes Metab. Disord..

[60] Hajifaraji M., Jahanjou F., Abbasalizadeh F., Aghamohammadzadeh N., Abbasi M.M., Dolatkhah N. (2017). Effect of Probiotic Supplementation on Blood Pressure of Females with Gestational Diabetes Mellitus: A Randomized Double Blind Controlled Clinical Trial. Iran. Red. Crescent Med. J..

[61] Sperry M.F., Silva H.L.A., Balthazar C.F., Esmerino E.A., Verruck S., Prudencio E.S., Neto R.P.C., Tavares M.I.B., Peixoto J.C., Nazzaro F. (2018). Probiotic Minas Frescal Cheese Added with L. Casei 01: Physicochemical and Bioactivity Characterization and Effects on Hematological/Biochemical Parameters of Hypertensive Overweighted Women–A Randomized Double-Blind Pilot Trial. J. Funct. Foods.

[62] Romão Da Silva L.D.F., De Oliveira Y., De Souza E.L., De Luna Freire M.O., Braga V.D.A., Magnani M., De Brito Alves J.L. (2020). Effects of Probiotic Therapy on Cardio-Metabolic Parameters and Autonomic Modulation in Hypertensive Women: A Randomized, Triple-Blind, Placebo-Controlled Trial. Food Funct..

[63] Xu J., Lazou Ahrén I., Olsson C., Jeppsson B., Ahrné S., Molin G. (2015). Oral and Faecal Microbiota in Volunteers with Hypertension in a Double Blind, Randomised Placebo Controlled Trial with Probiotics and Fermented Bilberries. J. Funct. Foods.

[64] Ivey K.L., Hodgson J.M., Kerr D.A., Thompson P.L., Stojceski B., Prince R.L. (2015). The Effect of Yoghurt and Its Probiotics on Blood Pressure and Serum Lipid Profile; a Randomised Controlled Trial. Nutr. Metab. Cardiovasc. Dis..

[65] Faselis C., Katsimardou A., Imprialos K., Deligkaris P., Kallistratos M., Dimitriadis K. (2019). Microvascular Complications of Type 2 Diabetes Mellitus. Curr. Vasc. Pharmacol..

[66] Tanase D.M., Gosav E.M., Neculae E., Costea C.F., Ciocoiu M., Hurjui L.L., Tarniceriu C.C., Maranduca M.A., Lacatusu C.M., Floria M. (2020). Role of Gut Microbiota on Onset and Progression of Microvascular Complications of Type 2 Diabetes (T2DM). Nutrients.

[67] Leenders F., Groen N., de Graaf N., Engelse M.A., Rabelink T.J., de Koning E.J.P., Carlotti F. (2021). Oxidative Stress Leads to β-Cell Dysfunction through Loss of β-Cell Identity. Front. Immunol..

[68] Robertson R.P., Harmon J., Tran P.O.T., Poitout V. (2004). β-Cell Glucose Toxicity, Lipotoxicity, and Chronic Oxidative Stress in Type 2 Diabetes. Diabetes.

[69] Aw W., Fukuda S. (2018). Understanding the Role of the Gut Ecosystem in Diabetes Mellitus. J. Diabetes Investig..

[70] Gurung M., Li Z., You H., Rodrigues R., Jump D.B., Morgun A., Shulzhenko N. (2020). Role of Gut Microbiota in Type 2 Diabetes Pathophysiology. EBioMedicine.

[71] Miraghajani M., Zaghian N., Mirlohi M., Feizi A., Ghiasvand R. (2017). The Impact of Probiotic Soy Milk Consumption on Oxidative Stress Among Type 2 Diabetic Kidney Disease Patients: A Randomized Controlled Clinical Trial. J. Ren. Nutr..

[72] Feizollahzadeh S., Ghiasvand R., Rezaei A., Khanahmad H., Sadeghi A., Hariri M. (2017). Effect of Probiotic Soy Milk on Serum Levels of Adiponectin, Inflammatory Mediators, Lipid Profile, and Fasting Blood Glucose Among Patients with Type II Diabetes Mellitus. Probiotics Antimicrob. Proteins.

[73] Miraghajani M., Zaghian N., Dehkohneh A., Mirlohi M., Ghiasvand R. (2019). Probiotic Soy Milk Consumption and Renal Function Among Type 2 Diabetic Patients with Nephropathy: A Randomized Controlled Clinical Trial. Probiotics Antimicrob. Proteins.

[74] Sato J., Kanazawa A., Azuma K., Ikeda F., Goto H., Komiya K., Kanno R., Tamura Y., Asahara T., Takahashi T. (2017). Probiotic Reduces Bacterial Translocation in Type 2 Diabetes Mellitus: A Randomised Controlled Study. Sci. Rep..

[75] Sabico S., Al-Mashharawi A., Al-Daghri N.M., Yakout S., Alnaami A.M., Alokail M.S., McTernan P.G. (2017). Effects of a Multi-Strain Probiotic Supplement for 12 Weeks in Circulating Endotoxin Levels and Cardiometabolic Profiles of Medication Naïve T2DM Patients: A Randomized Clinical Trial. J. Transl. Med..

[76] Mobini R., Tremaroli V., Ståhlman M., Karlsson F., Levin M., Ljungberg M., Sohlin M., Bertéus Forslund H., Perkins R., Bäckhed F. (2017). Metabolic Effects of *Lactobacillus reuteri* DSM 17938 in People with Type 2 Diabetes: A Randomized Controlled Trial. Diabetes Obes. Metab..

[77] Kobyliak N., Falalyeyeva T., Mykhalchyshyn G., Kyriienko D., Komissarenko I. (2018). Effect of Alive Probiotic on Insulin Resistance in Type 2 Diabetes Patients: Randomized Clinical Trial. Diabetes Metab. Syndr. Clin. Res. Rev..

[78] Sabico S., Al-Mashharawi A., Al-Daghri N.M., Wani K., Amer O.E., Hussain D.S., Ahmed Ansari M.G., Masoud M.S., Alokail M.S., McTernan P.G. (2019). Effects of a 6-Month Multi-Strain Probiotics Supplementation in Endotoxemic, Inflammatory and Cardiometabolic Status of T2DM Patients: A Randomized, Double-Blind, Placebo-Controlled Trial. Clin. Nutr..

[79] Razmpoosh E., Javadi A., Ejtahed H.S., Mirmiran P., Javadi M., Yousefinejad A. (2019). The Effect of Probiotic Supplementation on Glycemic Control and Lipid Profile in Patients with Type 2 Diabetes: A Randomized Placebo Controlled Trial. Diabetes Metab. Syndr. Clin. Res. Rev..

[80] Khalili L., Alipour B., Jafar-Abadi M.A., Faraji I., Hassanalilou T., Abbasi M.M., Vaghef-Mehrabany E., Sani M.A. (2019). The Effects of *Lactobacillus casei* on Glycemic Response, Serum Sirtuin1 and Fetuin-A Levels in Patients with Type 2 Diabetes Mellitus: A Randomized Controlled Trial. Iran. Biomed. J..

[81] Madempudi R.S., Ahire J.J., Neelamraju J., Tripathi A., Nanal S. (2019). Efficacy of UB0316, a Multi-Strain Probiotic Formulation in Patients with Type 2 Diabetes Mellitus: A Double Blind, Randomized, Placebo Controlled Study. PLoS ONE.

[82] Ahmadian F., Razmpoosh E., Ejtahed H.S., Javadi M., Mirmiran P., Azizi F. (2022). Effects of Probiotic Supplementation on Major Cardiovascular-Related Parameters in Patients with Type-2 Diabetes Mellitus: A Secondary-Data Analysis of a Randomized Double-Blind Controlled Trial. Diabetol. Metab. Syndr..

[83] Tao Y.W., Gu Y.L., Mao X.Q., Zhang L., Pei Y.F. (2020). Effects of Probiotics on Type II Diabetes Mellitus: A Meta-Analysis. J. Transl. Med..

[84] Rehman K., Akash M.S.H. (2017). Mechanism of Generation of Oxidative Stress and Pathophysiology of Type 2 Diabetes Mellitus: How Are They Interlinked?. J. Cell. Biochem..

[85] Dinarello C.A., Donath M.Y., Mandrup-Poulsen T. (2010). Role of IL-1β in Type 2 Diabetes. Curr. Opin. Endocrinol. Diabetes Obes..

[86] Oguntibeju O.O. (2019). Type 2 Diabetes Mellitus, Oxidative Stress and Inflammation: Examining the Links. Int. J. Physiol. Pathophysiol. Pharmacol..

[87] Aleidi S., Issa A., Bustanji H., Khalil M., Bustanji Y. (2015). Adiponectin Serum Levels Correlate with Insulin Resistance in Type 2 Diabetic Patients. Saudi Pharm. J..

[88] Moonishaa T., Nanda S., Shamraj M., Sivaa R., Sivakumar P., Ravichandran K. (2017). Evaluation of Leptin as a Marker of Insulin Resistance in Type 2 Diabetes Mellitus. Int. J. Appl. Basic. Med. Res..

[89] Mooradian A.D. (2009). Dyslipidemia in Type 2 Diabetes Mellitus. Nat. Clin. Pract. Endocrinol. Metab..

[90] Tune J.D., Goodwill A.G., Sassoon D.J., Mather K.J. (2017). Cardiovascular Consequences of Metabolic Syndrome. Transl. Res..

[91] Noubiap J.J., Nansseu J.R., Lontchi-Yimagou E., Nkeck J.R., Nyaga U.F., Ngouo A.T., Tounouga D.N., Tianyi F.L., Foka A.J., Ndoadoumgue A.L. (2022). Geographic Distribution of Metabolic Syndrome and Its Components in the General Adult Population: A Meta-Analysis of Global Data from 28 Million Individuals. Diabetes Res. Clin. Pract..

[92] Cleeman J.I. (2001). Executive Summary of the Third Report of the National Cholesterol Education Program (NCEP) Expert Panel on Detection, Evaluation, and Treatment of High Blood Cholesterol in Adults (Adult Treatment Panel III). J. Am. Med. Assoc..

[93] Castro-Barquero S., Ruiz-León A.M., Sierra-Pérez M., Estruch R., Casas R. (2020). Dietary Strategies for Metabolic Syndrome: A Comprehensive Review. Nutrients.

[94] Hoyas I., Leon-Sanz M. (2019). Nutritional Challenges in Metabolic Syndrome. J. Clin. Med..

[95] Xavier-Santos D., Bedani R., Lima E.D., Saad S.M.I. (2020). Impact of Probiotics and Prebiotics Targeting Metabolic Syndrome. J. Funct. Foods.

[96] Rezazadeh L., Gargari B.P., Jafarabadi M.A., Alipour B. (2019). Effects of Probiotic Yogurt on Glycemic Indexes and Endothelial Dysfunction Markers in Patients with Metabolic Syndrome. Nutrition.

[97] Rezazadeh L., Alipour B., Jafarabadi M.A., Behrooz M., Gargari B.P. (2021). Daily Consumption Effects of Probiotic Yogurt Containing *Lactobacillus acidophilus* La5 and *Bifidobacterium lactis* Bb12 on Oxidative Stress in Metabolic Syndrome Patients. Clin. Nutr. ESPEN.

[98] Ghizi A.C.d.S., de Almeida Silva M., Moraes F.S.d.A., da Silva C.L., Endringer D.C., Scherer R., Lenz D., de Lima E.M., Brasil G.A., Maia J.F. (2021). Kefir Improves Blood Parameters and Reduces Cardiovascular Risks in Patients with Metabolic Syndrome. PharmaNutrition.

[99] Bellikci-Koyu E., Sarer-Yurekli B.P., Akyon Y., Aydin-Kose F., Karagozlu C., Ozgen A.G., Brinkmann A., Nitsche A., Ergunay K., Yilmaz E. (2019). Effects of Regular Kefir Consumption on Gut Microbiota in Patients with Metabolic Syndrome: A Parallel-Group, Randomized, Controlled Study. Nutrients.

[100] Bellikci-Koyu E., Sarer-Yurekli B.P., Karagozlu C., Aydin-Kose F., Ozgen A.G., Buyuktuncer Z. (2022). Probiotic Kefir Consumption Improves Serum Apolipoprotein A1 Levels in Metabolic Syndrome Patients: A Randomized Controlled Clinical Trial. Nutr. Res..

[101] Tripolt N.J., Leber B., Triebl A., Köfeler H., Stadlbauer V., Sourij H. (2015). Effect of *Lactobacillus casei* Shirota Supplementation on Trimethylamine-N-Oxide Levels in Patients with Metabolic Syndrome: An Open-Label, Randomized Study. Atherosclerosis.

[102] Bernini L.J., Simão A.N.C., Alfieri D.F., Lozovoy M.A.B., Mari N.L., de Souza C.H.B., Dichi I., Costa G.N. (2016). Beneficial Effects of *Bifidobacterium lactis* on Lipid Profile and Cytokines in Patients with Metabolic Syndrome: A Randomized Trial. Effects of Probiotics on Metabolic Syndrome. Nutrition.

[103] Bernini L.J., Simão A.N.C., De Souza C.H.B., Alfieri D.F., Segura L.G., Costa G.N., Dichi I. (2018). Effect of *Bifidobacterium lactis* HN019 on Inflammatory Markers and Oxidative Stress in Subjects with and without the Metabolic Syndrome. Br. J. Nutr..

[104] Kazemi A., Noorbala A.A., Azam K., Djafarian K. (2019). Effect of Prebiotic and Probiotic Supplementation on Circulating Pro-Inflammatory Cytokines and Urinary Cortisol Levels in Patients with Major Depressive Disorder: A Double-Blind, Placebo-Controlled Randomized Clinical Trial. J. Funct. Foods.

[105] Xiao R., Chen Y., Zhu X., Wang L., Tian P., Jin X., Liang M., Chen Z., Zhang T., Qian L. (2024). A Randomised Double-Blind Placebo-Controlled Trial of a Probiotic Combination for Manipulating the Gut Microbiota and Managing Metabolic Syndrome. Food Biosci..

[106] Babio N., Martínez-González M.A., Estruch R., Wärnberg J., Recondo J., Ortega-Calvo M., Serra-Majem L., Corella D., Fitó M., Ros E. (2015). Associations between Serum Uric Acid Concentrations and Metabolic Syndrome and Its Components in the PREDIMED Study. Nutr. Metab. Cardiovasc. Dis..

[107] Sreckovic B., Sreckovic V.D., Soldatovic I., Colak E., Sumarac-Dumanovic M., Janeski H., Janeski N., Gacic J., Mrdovic I. (2017). Homocysteine Is a Marker for Metabolic Syndrome and Atherosclerosis. Diabetes Metab. Syndr. Clin. Res. Rev..

[108] Kumari R., Kumar S., Kant R. (2019). An Update on Metabolic Syndrome: Metabolic Risk Markers and Adipokines in the Development of Metabolic Syndrome. Diabetes Metab. Syndr. Clin. Res. Rev..

[109] Barrea L., Annunziata G., Muscogiuri G., Di Somma C., Laudisio D., Maisto M., de Alteriis G., Tenore G.C., Colao A., Savastano S. (2018). Trimethylamine-N-Oxide (TMAO) as Novel Potential Biomarker of Early Predictors of Metabolic Syndrome. Nutrients.

[110] Mach F., Baigent C., Catapano A.L., Koskina K.C., Casula M., Badimon L., Chapman M.J., De Backer G.G., Delgado V., Ference B.A. (2019). 2019 ESC/EAS Guidelines for the Management of Dyslipidaemias: Lipid Modification to Reduce Cardiovascular Risk. Atherosclerosis.

[111] Civeira F., Arca M., Cenarro A., Hegele R.A. (2022). A Mechanism-Based Operational Definition and Classification of Hypercholesterolemia. J. Clin. Lipidol..

[112] Bhatnagar D., Soran H., Durrington P.N. (2008). Hypercholesterolaemia and Its Management. BMJ.

[113] El-Tantawy W.H., Temraz A. (2019). Natural Products for Controlling Hyperlipidemia: Review. Arch. Physiol. Biochem..

[114] De Ferranti S.D., Rodday A.M., Mendelson M.M., Wong J.B., Leslie L.K., Sheldrick R.C. (2016). Prevalence of Familial Hypercholesterolemia in the 1999 to 2012 United States National Health and Nutrition Examination Surveys (NHANES). Circulation.

[115] Hunter P.M., Hegele R.A. (2017). Functional Foods and Dietary Supplements for the Management of Dyslipidaemia. Nat. Rev. Endocrinol..

[116] Tsai C.C., Lin P.P., Hsieh Y.M., Zhang Z.Y., Wu H.C., Huang C.C. (2014). Cholesterol-Lowering Potentials of Lactic Acid Bacteria Based on Bile-Salt Hydrolase Activity and Effect of Potent Strains on Cholesterol Metabolism In Vitro and In Vivo. Sci. World J..

[117] Ma C., Zhang S., Lu J., Zhang C., Pang X., Lv J. (2019). Screening for Cholesterol-Lowering Probiotics from Lactic Acid Bacteria Isolated from Corn Silage Based on Three Hypothesized Pathways. Int. J. Mol. Sci..

[118] Chiu H.F., Fang C.Y., Shen Y.C., Venkatakrishnan K., Wang C.K. (2021). Efficacy of Probiotic Milk Formula on Blood Lipid and Intestinal Function in Mild Hypercholesterolemic Volunteers: A Placebo-Control, Randomized Clinical Trial. Probiotics Antimicrob. Proteins.

[119] Costabile A., Buttarazzi I., Kolida S., Quercia S., Baldini J., Swann J.R., Brigidi P., Gibson G.R. (2017). An in Vivo Assessment of the Cholesterol-Lowering Efficacy of *Lactobacillus plantarum* ECGC 13110402 in Normal to Mildly Hypercholesterolaemic Adults. PLoS ONE.

[120] Khongrum J., Yingthongchai P., Boonyapranai K., Wongtanasarasin W., Aobchecy P., Tateing S., Prachansuwan A., Sitdhipol J., Niwasabutra K., Thaveethaptaikul P. (2023). Safety and Effects of *Lactobacillus paracasei* TISTR 2593 Supplementation on Improving Cholesterol Metabolism and Atherosclerosis-Related Parameters in Subjects with Hypercholesterolemia: A Randomized, Double-Blind, Placebo-Controlled Clinical Trial. Nutrients.

[121] Ryan J.J., Hanes D.A., Schafer M.B., Mikolai J., Zwickey H. (2015). Effect of the Probiotic *Saccharomyces boulardii* on Cholesterol and Lipoprotein Particles in Hypercholesterolemic Adults: A Single-Arm, Open-Label Pilot Study. J. Altern. Complement. Med..

[122] Martoni C.J., Labbe A., Ganopolsky J.G., Prakash S., Jones M.L. (2015). Changes in Bile Acids, FGF-19 and Sterol Absorption in Response to Bile Salt Hydrolase Active *L. reuteri* NCIMB 30242. Gut Microbes.

[123] Ito M., Kusuhara S., Yokoi W., Sato T., Ishiki H., Miida S., Matsui A., Nakamori K., Nonaka C., Miyazaki K. (2017). *Streptococcus thermophilus* Fermented Milk Reduces Serum MDA-LDL and Blood Pressure in Healthy and Mildly Hypercholesterolaemic Adults. Benef. Microbes.

[124] Kumar M., Nagpal R., Kumar R., Hemalatha R., Verma V., Kumar A., Chakraborty C., Singh B., Marotta F., Jain S. (2012). Cholesterol-lowering Probiotics as Potential Biotherapeutics for Metabolic Diseases. J. Diabetes Res..

[125] Juste C., Gérard P. (2021). Cholesterol-to-Coprostanol Conversion by the Gut Microbiota: What We Know, Suspect, and Ignore. Microorganisms.

[126] Liong M.T., Shah N.P. (2005). Bile Salt Deconjugation and BSH Activity of Five *Bifidobacterial* Strains and Their Cholesterol Co-Precipitating Properties. Food Res. Int..

[127] Yao Y., Hong Q., Ding S., Cui J., Li W., Zhang J., Sun Y., Yu Y., Yu M., Mi L. (2024). Meta-Analysis of the Effects of Probiotics on Hyperlipidemia. Curr. Res. Food Sci..

[128] Collado A., Marques P., Domingo E., Perello E., González-Navarro H., Martinez-Hervás S., Real J.T., Piqueras L., Ascaso J.F., Sanz M.J. (2019). Novel Immune Features of the Systemic Inflammation Associated with Primary Hypercholesterolemia: Changes in Cytokine/Chemokine Profile, Increased Platelet and Leukocyte Activation. J. Clin. Med..

[129] Cristofori F., Dargenio V.N., Dargenio C., Miniello V.L., Barone M., Francavilla R. (2021). Anti-Inflammatory and Immunomodulatory Effects of Probiotics in Gut Inflammation: A Door to the Body. Front. Immunol..

[130] Malakar A.K., Choudhury D., Halder B., Paul P., Uddin A., Chakraborty S. (2019). A Review on Coronary Artery Disease, Its Risk Factors, and Therapeutics. J. Cell Physiol..

[131] Moran A.E., Oliver J.T., Mirzaie M., Forouzanfar M.H., Chilov M., Anderson L., Morrison J.L., Khan A., Zhang N., Haynes N. (2012). Assessing the Global Burden of Ischemic Heart Disease: Part 1: Methods for a Systematic Review of the Global Epidemiology of Ischemic Heart Disease in 1990 and 2010. Glob. Heart.

[132] Nowbar A.N., Howard J.P., Finegold J.A., Asaria P., Francis D.P. (2014). 2014 Global Geographic Analysis of Mortality from Ischaemic Heart Disease by Country, Age and Income: Statistics from World Health Organisation and United Nations. Int. J. Cardiol..

[133] Moludi J., Kafil H.S., Qaisar S.A., Gholizadeh P., Alizadeh M., Vayghyan H.J. (2021). Effect of Probiotic Supplementation along with Calorie Restriction on Metabolic Endotoxemia, and Inflammation Markers in Coronary Artery Disease Patients: A Double Blind Placebo Controlled Randomized Clinical Trial. Nutr. J..

[134] Sun B., Ma T., Li Y., Yang N., Li B., Zhou X., Guo S., Zhang S., Kwok L.-Y., Sun Z. (2022). *Bifidobacterium lactis* Probio-M8 Adjuvant Treatment Confers Added Benefits to Patients with Coronary Artery Disease via Target Modulation of the Gut-Heart/-Brain Axes. mSystems.

[135] Raygan F., Rezavandi Z., Bahmani F., Ostadmohammadi V., Mansournia M.A., Tajabadi-Ebrahimi M., Borzabadi S., Asemi Z. (2018). The Effects of Probiotic Supplementation on Metabolic Status in Type 2 Diabetic Patients with Coronary Heart Disease IRCT2017082733941N5 IRCT. Diabetol. Metab. Syndr..

[136] Malik M., Suboc T.M., Tyagi S., Salzman N., Wang J., Ying R., Tanner M.J., Kakarla M., Baker J.E., Widlansky M.E. (2018). *Lactobacillus plantarum* 299v Supplementation Improves Vascular Endothelial Function and Reduces Inflammatory Biomarkers in Men with Stable Coronary Artery Disease. Circ. Res..

[137] Hofeld B.C., Puppala V.K., Tyagi S., Ahn K.W., Anger A., Jia S., Salzman N.H., Hessner M.J., Widlansky M.E. (2021). *Lactobacillus plantarum* 299v Probiotic Supplementation in Men with Stable Coronary Artery Disease Suppresses Systemic Inflammation. Sci. Rep..

[138] Moludi J., Khedmatgozar H., Nachvak S.M., Abdollahzad H., Moradinazar M., Tabaei A.S. (2022). The Effects of Co-Administration of Probiotics and Prebiotics on Chronic Inflammation, and Depression Symptoms in Patients with Coronary Artery Diseases: A Randomized Clinical Trial. Nutr. Neurosci..

[139] Vinay B.C., Shastry C.S., Kodangala S., Mateti U.V., Bhat K. (2020). Association of Diet and Lipid Profile among Coronary Heart Disease Patients. Clin. Epidemiol. Glob. Health.

